# BAY-3827 and SBI-0206965: Potent AMPK Inhibitors That Paradoxically Increase Thr172 Phosphorylation

**DOI:** 10.3390/ijms25010453

**Published:** 2023-12-29

**Authors:** Simon A. Hawley, Fiona M. Russell, Fiona A. Ross, D. Grahame Hardie

**Affiliations:** Division of Cell Signalling & Immunology, School of Life Sciences, University of Dundee, Dow Street, Dundee DD1 5EH, UK; s.a.hawley@dundee.ac.uk (S.A.H.); fross001@dundee.ac.uk (F.A.R.)

**Keywords:** AMP-activated protein kinase, AMPK, BAY-3827, cancer, compound C, kinase inhibitors, LKB1, SBI-0206965

## Abstract

AMP-activated protein kinase (AMPK) is the central component of a signalling pathway that senses energy stress and triggers a metabolic switch away from anabolic processes and towards catabolic processes. There has been a prolonged focus in the pharmaceutical industry on the development of AMPK-activating drugs for the treatment of metabolic disorders such as Type 2 diabetes and non-alcoholic fatty liver disease. However, recent findings suggest that AMPK inhibitors might be efficacious for treating certain cancers, especially lung adenocarcinomas, in which the *PRKAA1* gene (encoding the α1 catalytic subunit isoform of AMPK) is often amplified. Here, we study two potent AMPK inhibitors, BAY-3827 and SBI-0206965. Despite not being closely related structurally, the treatment of cells with either drug unexpectedly caused increases in AMPK phosphorylation at the activating site, Thr172, even though the phosphorylation of several downstream targets in different subcellular compartments was completely inhibited. Surprisingly, the two inhibitors appear to promote Thr172 phosphorylation by different mechanisms: BAY-3827 primarily protects against Thr172 dephosphorylation, while SBI-0206965 also promotes phosphorylation by LKB1 at low concentrations, while increasing cellular AMP:ATP ratios at higher concentrations. Due to its greater potency and fewer off-target effects, BAY-3827 is now the inhibitor of choice for cell studies, although its low bioavailability may limit its use in vivo.

## 1. Introduction

The AMP-activated protein kinase (AMPK) is the core component of an ancient signalling pathway that acts as a sensor of cellular nutrient and energy status and opposes the growth-promoting mTORC1 pathway [1,2,3]. AMPK is expressed in essentially all eukaryotic cells as heterotrimeric complexes comprising catalytic α subunits and regulatory β and γ subunits. In mammals, the three subunits are encoded by multiple genes, giving rise to seven subunit isoforms (α1, α2; β1, β2; and γ1, γ2, and γ3) that can generate up to twelve heterotrimeric combinations. Stresses that either inhibit ATP production from ADP, or that elevate ATP breakdown to ADP, increase the cellular ADP:ATP ratio, which is amplified by the adenylate kinase reaction into even larger increases in AMP:ATP ratio. These changes are sensed by the competitive binding of AMP and ATP at CBS3, one of three adenine nucleotide-binding sites formed by the tandem CBS repeats on the AMPK-γ subunits [4,5]. The replacement of ATP by AMP at CBS3 triggers a major conformational change [6,7], causing the activation of AMPK through three complementary mechanisms: (i) the promotion of phosphorylation at Thr172 within the activation loop of the α subunit kinase domain by the upstream kinase, LKB1 [8,9], causing up to 100-fold activation [10]; (ii) inhibition of the dephosphorylation of Thr172 by protein phosphatases [8]; and (iii) the allosteric activation of AMPK complexes already phosphorylated on Thr172, causing further activation [8,11]. AMPK then phosphorylates downstream targets at serine or threonine residues lying within a well-defined recognition motif, with at least 100 direct targets currently identified [3]. As well as this canonical energy-sensing role, AMPK is also activated by various non-canonical (AMP-independent) pathways [3].

Findings that AMPK activation enhanced muscle glucose uptake [12] motivated the development of screens that searched for pharmacological activators of AMPK, resulting in the identification of several allosteric activators, including A-769662 [13], MK-8722 [14], PF-739 [15], and PXL-770 [16]. These compounds all bind in a cleft called the Allosteric Drug and Metabolite (ADaM) site [17] located between the α and β subunits [18]. They are all synthetic compounds and not natural products, but CoA esters of medium- to long-chain fatty acids also activate AMPK by binding at the ADaM site [19].

Although most interest in AMPK within the pharmaceutical industry has focused on activators, recent studies on the role of AMPK in cancer have triggered interest in inhibitors [20]. Studies on a mouse model of T cell acute lymphoblastic leukaemia/lymphoma (T-ALL) showed that T cell-specific knockout of the *Prkaa1* gene (encoding AMPK-α1, the sole catalytic subunit isoform in T cells) led to earlier onset and more rapid development of T-ALL triggered by *Pten* knockout, showing that AMPK can act as a tumour suppressor [21]. By contrast, in another T-ALL model involving murine T cell progenitor cells transformed in vitro and then grown as allografts in recipient mice, the subsequent knockout of *Prkaa1* led to reduced recovery of T-ALL cells and enhanced survival of the mice, suggesting that AMPK-α1 was acting as a tumour promoter [22]. The key difference between these two models is that, in the first, AMPK was knocked out prior to the development of T-ALL, while, in the second, AMPK was knocked out after disease had become established. Thus, while AMPK activators (if given before cancer arises) might be useful in reducing its initiation in at-risk individuals, AMPK inhibitors are more likely to be useful in treating pre-existing cancers.

Similar dichotomies are evident from consideration of human cancers. Analyses of genetic changes in AMPK genes in cancer genome studies [20,23] show that, while *PRKAA2* (encoding AMPK-α2) exhibits numerous mis-sense mutations in skin cancers (which might be expected of a tumour suppressor), the *PRKAA1* gene is frequently amplified in lung cancers, more consistent with AMPK-α1 being a tumour promoter. Indeed, AMPK inhibitors might be particularly useful in treating cancers where the *PRKAA1* gene is amplified, which include at least 10% of all cases of human lung adenocarcinoma [20,23].

The first AMPK inhibitor to be developed was compound C [24], also known as dorsomorphin [25]. On the basis of screening against just five other protein kinases, it was claimed to be a selective inhibitor of AMPK [24]. However, in a screen against 71 kinases, 9 were inhibited by 1 µM of compound C to a greater extent than AMPK [26], while in a screen of 110 kinases [27], no less than 30 were inhibited by 10 µM of compound C to a greater extent than AMPK (Supplementary Figure S1A). Thus, compound C is not a selective inhibitor of AMPK and should not be used for that purpose.

SBI-0206965 (Figure S2), originally developed as an inhibitor of the kinase ULK1 [28], was later shown to inhibit AMPK with a similar potency [29]. Although it does inhibit ULK1 and ULK2 and a few other kinases (Figure S1B), it is much more selective for AMPK than compound C (Figure S1A). In primary mouse hepatocytes, SBI-0206965 inhibited 991-induced ACC phosphorylation and promoted fatty acid synthesis as expected for an AMPK inhibitor, although studies in C2C12 myotubes and cell-free screens have also revealed AMPK-independent, off-target effects [30].

More recently, a novel AMPK inhibitor, BAY-3827 (Figure S2), was found to be efficacious in inhibiting the proliferation of prostate cancer cell lines [31] (further information about BAY-3827 is available on the Structural Genomics Consortium website [32]). Using low ATP concentrations (10 µM) in assays, BAY-3827 inhibited human AMPK (α2β1γ1 complex) with a remarkably low IC_50_ of 1.4 nM. A control compound (BAY-974, Figure S2) that did not inhibit AMPK, despite having a very similar structure to BAY-3827, was also described. AMPK-α1 or -α2 were among the kinases most potently inhibited by BAY-3827, and a selectivity screen against 331 kinases revealed that BAY-3827 had even better selectivity for AMPK than SBI-0206965, although it did potently inhibit all four isoforms of RSK (RSK1-4, also known as RPS6KA1-4, Figure S1A,C). However, little or no information about the mechanism of AMPK inhibition by BAY-3827 has been provided [31,32]. In the present study, we characterize the mechanism of the inhibition of AMPK by BAY-3827, and report that it paradoxically enhances the phosphorylation of AMPK at the activating site, Thr172, by inhibiting its dephosphorylation. Interestingly SBI-0206965, although not structurally related, exhibited the same paradoxical effect, although the detailed mechanism(s) appears to be different. Since Thr172 phosphorylation is often used as a surrogate marker for AMPK activity, these paradoxical increases in Thr172 phosphorylation by potent AMPK inhibitors need to be taken into account when using these compounds in intact cells.

## 2. Results

### 2.1. BAY-3827 Is a Mixed Type Inhibitor of AMPK

**Figure 1:**
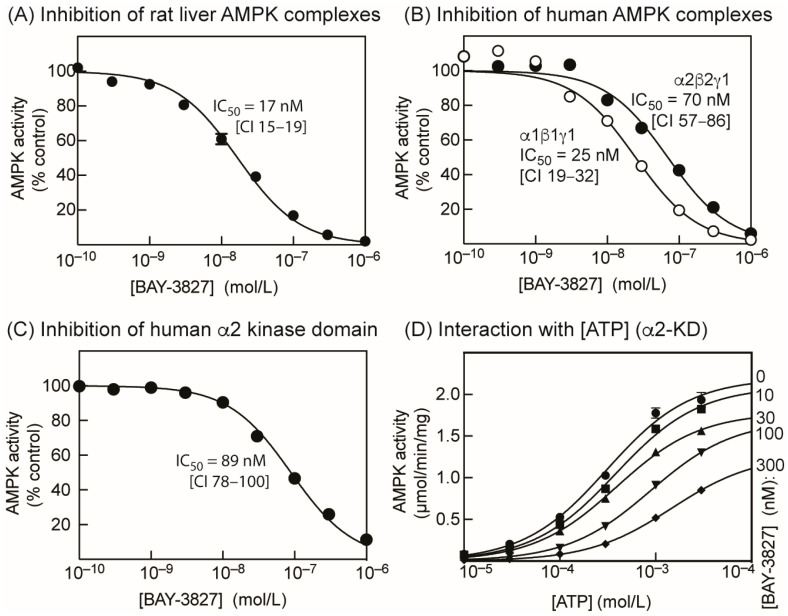
Effects of BAY-3827 on preparations of purified AMPK in cell-free assays. (**A**) Inhibition of rat liver AMPK by increasing concentrations of BAY-3827. Symbols represent the mean (±SD) of the actual data (*n* = 2), while the continuous curve was obtained by fitting to the equation Y = 100 − (100 × X)/(IC_50_ + X), where IC_50_ is the concentration of BAY-3827 giving a half-maximal effect; values for IC_50_ and 95% confidence interval (CI) are shown. (**B**) As (**A**) but using bacterially expressed human α1β1γ1 or α2β2γ1 complexes that had been maximally phosphorylated on Thr172 by CaMKK2. (**C**) As (**A**) but using a bacterially expressed glutathione-S-transferase (GST) fusion of the α2 kinase domain sequence (1–310) that had been maximally phosphorylated on Thr172 by CaMKK2. (**D**) Results of assays using the α2 kinase domain (as in (**C**)) with various concentrations of ATP in the presence of varying concentrations of BAY-3827. Mg^2+^ was maintained at a constant excess of 4.8 mM over ATP, a design that ensures that the concentration of the Mg.ATP^2−^ complex varies as a fixed proportion of total ATP [33]. For each concentration of BAY-3827, results (mean ± SD, *n* = 2) were fitted to the Michaelis–Menten equation [Y = Vmax × X/(Km + X)]. The continuous curves were plotted using this equation, using the best-fit values for Vmax and Km shown in Table 1.

Using the standard assay ATP concentration (200 µM), BAY-3827 inhibited native AMPK purified from rat liver (a mixture of the α1 and α2 catalytic subunit isoforms, primarily in complex with β1 and γ1) with an IC_50_ of 17 nM (Figure 1A). Using bacterially expressed human α1β1γ1 and α2β2γ1 complexes (phosphorylated on Thr172 by CaMKK2) in the same assay, the IC_50_ values were 25 and 70 nM, respectively (Figure 1B). We also tested the compound on the isolated kinase domain of the human α2 isoform (residues 1–310, phosphorylated on Thr172 by CaMKK2) and observed an IC_50_ of 89 nM (Figure 1C). Figure S3 shows plots equivalent to Figure 1A–C for inhibition by SBI-0206965 instead of BAY-3827. SBI-0206965 was between 5- and 20-fold less potent as an AMPK inhibitor than BAY-3827 in cell-free kinase assays.

When the ATP concentration in the assay was varied from 10 µM to 3 mM, increasing concentrations of BAY-3827 up to 300 nM progressively increased the apparent Km for MgATP of the isolated α2 kinase domain from 309 µM to 1.48 mM, while, at the same time, reducing the apparent Vmax from 2.2 to 1.3 µmol/min/mg (Figure 1D, Table 1). This indicates a mixed (competitive and uncompetitive) type of inhibition, similar to that observed previously for SBI-0206965 [29], which is classed as a Type II kinase inhibitor. Type II inhibitors occupy the adenosine pocket of the active site, but their binding also requires a specific, inactive conformation of the kinase domain, especially of the aspartate side chain in the DFG motif at the start of the activation loop [34].

### 2.2. BAY-3827 Interacts with Activation by AMP and MK-8722

**Figure 2:**
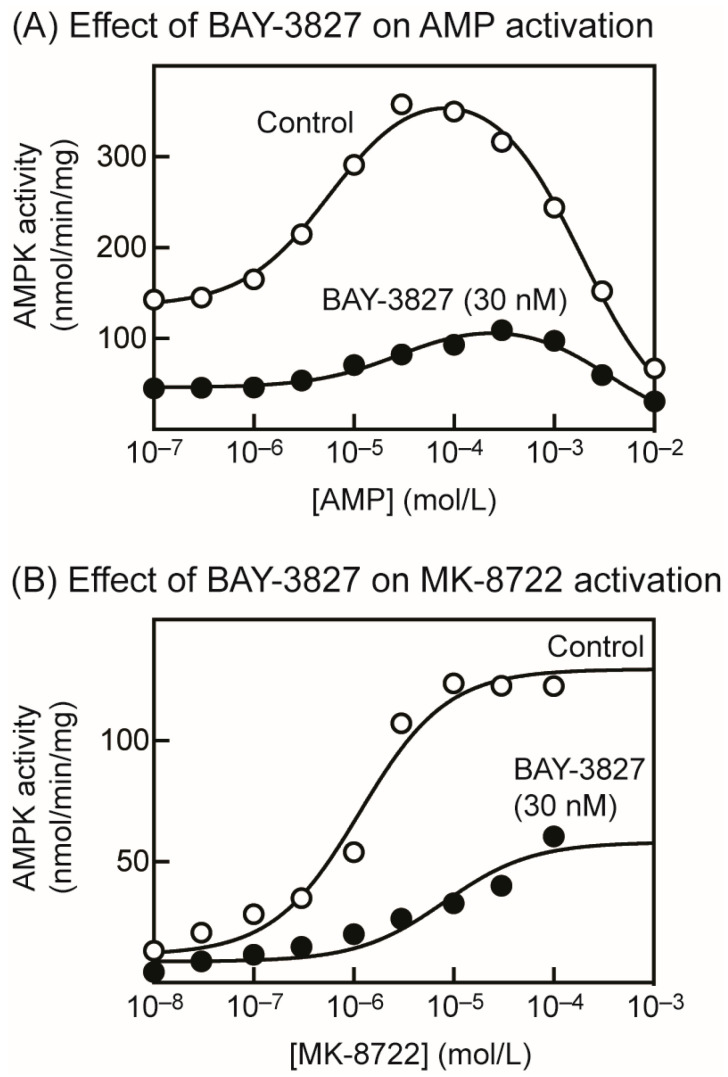
Interaction between inhibition of AMPK by BAY-3827 and allosteric activation by AMP or MK-8722. (**A**) Activation and inhibition of purified rat liver AMPK by increasing concentrations of AMP in the presence and absence of 30 nM BAY-3827. Results (mean ± SD, *n* = 2) were fitted to the equation: Y = Basal + (((Activation × Basal – Basal) × X)/(EC_50_ + X)) − (((Activation × Basal) × X)/(IC_50_ + X)) where Basal is the activity without AMP, Activation is the estimated maximal activation by AMP, and EC_50_ and IC_50_ are the concentrations of AMP causing half-maximal activation and inhibition, respectively. The continuous curves were obtained by plotting this equation using the best-fit values for Basal, Activation, EC_50_, and IC_50_ mentioned in the main text. (**B**) Activation of purified, “naïve” (i.e., unphosphorylated), bacterially expressed α1β1γ1 complex by increasing concentrations of MK-8722 in the presence and absence of 30 nM BAY-3827. Results (mean ± SD, *n* = 2) were fitted to the equation Y = Basal + ((Activation × Basal − Basal) × X)/(EC_50_ + X), where Basal is the basal activity without MK-8722, Activation is the estimated maximal activation by MK-8722, and EC_50_ is the concentration of MK-8722 causing half-maximal activation. The continuous curves were obtained by plotting this equation using the best-fit values for Basal, Activation, and EC_50_ mentioned in the main text.

Next, we studied the effect of BAY-3827 on the allosteric activation of rat liver AMPK by AMP (Figure 2A). Similar to previous results [8], low concentrations of AMP allosterically activated AMPK with an EC_50_ of 5.9 µM (CI 4.7–7.4), basal activity of 135 nmol·min^−1^·mg^−1^ (CI 127–143), and maximal activation of 2.9-fold (CI 2.7–3.1). At higher concentrations (above 100 µM), AMP then started to inhibit AMPK with an IC_50_ of 1.8 mM (CI 1.6–2.1); we have shown that this inhibitory effect, which occurs at AMP concentrations that are almost certainly too high to be physiologically relevant, is due to the competition of AMP with ATP at the catalytic site [8]. BAY-3827 (30 nM) inhibited at all concentrations of AMP, but also shifted the obtained bell-shaped curve to the right, i.e., to higher AMP concentrations (Figure 2A). The best-fit parameters were: basal activity of 46 nmol·min^−1^·mg^−1^ (CI 43–49), maximal activation of 2.7-fold (CI 2.4–2.9), EC_50_ of 30 µM (CI 19–52), and IC_50_ of 3.3 mM (CI 2.6–4.3). Thus, the EC_50_ increased 5-fold from 6 to 30 µM in the presence of BAY-3827.

We next looked for interactions between the effects of BAY-3827 and allosteric activation by the ADaM site activator, MK-8722 (Figure 2B). We used the “naïve” (unphosphorylated), bacterially expressed human α1β1γ1 complex, which displays a larger allosteric activation by another ADaM site activator, A-769662, than the same complex phosphorylated on Thr172 [35]. The maximal activation of the unphosphorylated α1β1γ1 complex by MK-8722 was 11-fold (CI 7.3–26), with basal activity of 11 nmol·min^−1^·mg^−1^ (CI 5–18) and EC_50_ of 1.1 µM (CI 0.79–1.6). Although BAY-3827 inhibited at all concentrations of MK-8722, not only was the maximal activation reduced (to 6.7-fold, CI 4.6–12), but the EC_50_ also increased 7-fold (to 8.1 µM, CI 2.4–27; basal activity 8.7 nmol·min^−1^·mg^−1^, CI 4.3–13).

### 2.3. Effects of BAY-3827 on Phosphorylation of AMPK Targets in U2OS Cells

**Figure 3:**
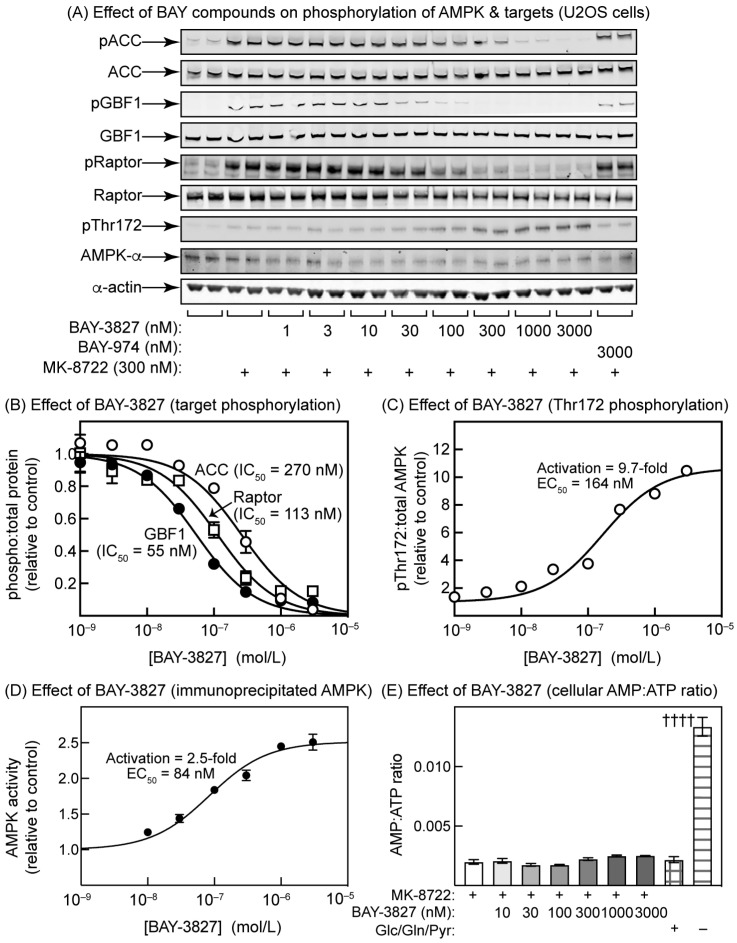
Effects of BAY-3827 on phosphorylation of AMPK and downstream targets in U2OS cells. (**A**) U2OS cells were incubated for 30 min with various concentrations of BAY-3827 or a single concentration (3 µM) of the inactive control compound, BAY-974, followed by 30 min with or without MK-8722 (300 nM). Lysates of duplicate dishes of cells were analysed with SDS-PAGE and resulting blots were probed with the phosphospecific and total protein antibodies/probes shown. The pACC antibody recognizes acetyl-CoA carboxylase (ACC) phosphorylated at Ser80. GBF1 is Golgi-specific brefeldin A resistance factor-1, which is associated with the Golgi apparatus and is phosphorylated by AMPK at Thr1337 [36], recognized by the phosphospecific antibody used. The pRaptor antibody recognizes Raptor phosphorylated at one of the AMPK sites (Ser792 [37]). (**B**) The blots in (**A**) were quantified using densitometry, expressed as ratios of signals obtained using phosphospecific and total protein antibodies/probes, and normalized to a value of 1.0 in the absence of inhibitor. Results (mean ± SD, *n* = 2) were fitted to the equation Y = 1 − (X)/(IC_50_ + X), where IC_50_ is the concentration of BAY-3827 causing half-maximal inhibition. The continuous curves were obtained by plotting this equation using the best-fit values for IC_50_ shown on the graph. (**C**) The blots in (**A**) obtained using the pThr172 and total AMPK-α antibodies were quantified using densitometry, expressed as ratios (pThr172:total AMPK) and normalized to a value of 1.0 in controls. Results (mean ± SD, *n* = 2) were fitted to the equation Y = 1 + (Activation × X)/(EC_50_ + X) where Activation is the increase in phosphorylation (relative to control) and EC_50_ is the concentration of BAY-3827 causing a half-maximal increase. The continuous curves were obtained by plotting this equation using the best-fit values for Activation and EC_50_ shown on the graph. (**D**) AMPK activity measured in anti-α1/-α2 immunoprecipitates from cells treated with different concentrations of BAY-3827, in a separate experiment to (**C**). Results are mean ± SEM (*n* = 3). Values for Activation and EC_50_ were determined as in (**C**) and the continuous line was drawn using the equation and those values. (**E**) Cellular AMP:ATP ratios estimated via LC:MS in an experiment parallel to that in (**D**). Results are mean ± SEM (*n* = 3). Daggers attached to the right-hand column show that the effect of starvation for 2 h of all exogenous carbon sources (glucose, glutamine, and pyruvate) was significantly different (*p* < 0.0001) from a control supplied with those carbon sources (adjacent column). However, none of the values in cells incubated with BAY-3827 were significantly different from the control with MK-8722 but without BAY-3827.

We next incubated human osteosarcoma (U2OS) cells with the ADaM site activator MK-8722, (which was added to increase the signals obtained with phosphospecific antibodies against AMPK targets), and various concentrations of BAY-3827 or the negative control compound, BAY-974. MK-8722 caused a markedly increased phosphorylation of three AMPK targets located in different subcellular compartments, i.e., acetyl-CoA carboxylase (ACC, Ser80, and cytosol), GBF1 (Thr1337 and Golgi complex), and Raptor (Ser792 and lysosome) (Figure 3A). All three phosphorylation events were progressively eliminated by increasing concentrations of BAY-3827, but not BAY-974; quantification of the effects of BAY-3827 on the ratio of the signals obtained with phosphospecific to the total antibodies/probes (Figure 3B) revealed that the IC_50_ values varied from 260 nM, CI 200–340 (ACC) to 113 nM, CI 85–150 (Raptor) and 55 nM, CI 46–64 (GBF1). Unexpectedly, the phosphorylation of Thr172 was increased by BAY-3827 (Figure 3A), up to a maximum of 9.7-fold (CI 8.8–11) with a half-maximal effect (EC_50_) at 164 nM (CI 110–240) (Figure 3C). This increase in Thr172 phosphorylation is paradoxical, because it would be expected to cause an increase in intrinsic AMPK activity. Indeed, when AMPK was immunoprecipated under conditions in which non-covalently bound ligands would be expected to be washed away but the phosphorylation of Thr172 preserved, AMPK was activated with an EC_50_ comparable with that for the effect on Thr172 phosphorylation, although the degree of activation was lower (Figure 3D). BAY-3827 did not cause any change in cellular AMP:ATP ratios, although in a positive control experiment in which the cells were starved of all carbon sources in the medium (glucose, glutamine, and pyruvate) for 2 h, there was a 6-fold increase in AMP:ATP ratio, as expected (Figure 3E).

### 2.4. SBI-0206965 Also Promotes Thr172 Phosphorylation in U2OS Cells

**Figure 4:**
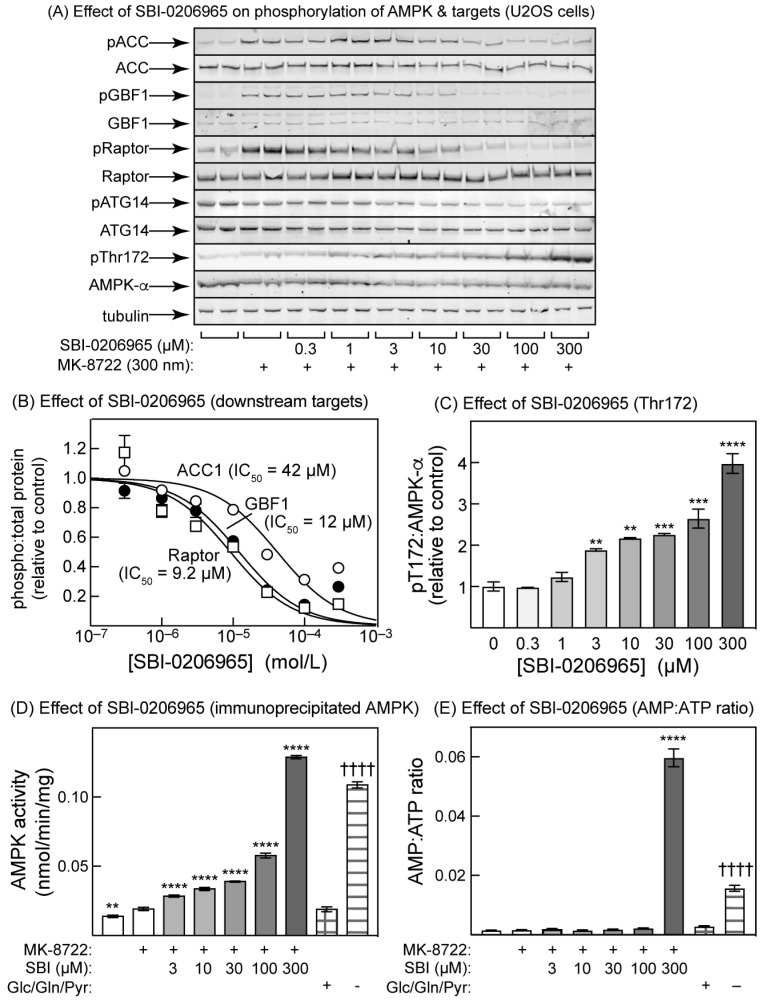
Effects of SBI-0206965 on phosphorylation of AMPK and downstream targets of AMPK and ULK1 in U2OS cells. (**A**) Cells were incubated for 30 min with the indicated concentrations of SBI-0206965, followed by 30 min with or without MK-8722 (300 nM). Duplicate lysates were analysed with SDS-PAGE and blots were probed with the indicated phosphospecific or total protein antibodies/probes. The antibodies/probes used were as in Figure 3, except that we also studied the effects on the ULK1 substrate ATG14 (Ser29). (**B**) The blots in (**A**) were quantified using densitometry, and the results obtained using the phosphospecific antibodies were expressed as a ratio of the results obtained using the total protein antibodies/probes (except for pGBF1 where the tubulin loading control was used instead) and normalized to a value of 1.0 in the absence of an inhibitor. Results (mean ± SD, *n* = 2) were fitted to the equation Y = 1 − (X)/(IC_50_ + X), where IC_50_ is the concentration of SBI-0206965 causing half-maximal inhibition. The continuous curves were obtained by plotting this equation using the best-fit values for IC_50_ shown on the graph. (**C**) The blots in (**A**) obtained using the pThr172 and total AMPK-α antibodies were quantified using densitometry, and the results obtained using anti-pThr172 were expressed as ratios of the results obtained using anti-AMPK-α antibody and normalized to a value of 1.0 in the absence of inhibitor. (**D**) Activation of AMPK by treatment of U2OS cells with MK-8722 and increasing concentrations of SBI-0206965. The experiment was as in (**A**), except that we omitted the two lowest concentrations of SBI-0206965, which had a negligible effect. As positive controls for AMPK activation by the canonical mechanism, we incubated cells for 2 h either with full medium containing 25 mM of glucose, 4 mM of glutamine, and 1 mM of pyruvate, or with medium lacking all three carbon sources (two right-hand columns). (**E**) As (**D**), but extracting cellular nucleotides with perchloric acid and measuring AMP:ATP ratios via LC:MS. In (**C**), asterisks signify mean values significantly different from the control without SBI-0206965. In (**D**,**E**), asterisks signify mean values significantly different from the control with MK-8722 but without SBI-0206965 (second column), while daggers signify mean values significantly different from the control with full medium (last column but one).

Modest increases in Thr172 phosphorylation in response to SBI-0206965 treatment of cells were evident in previous studies, but either were not commented upon [28], were ascribed to mitochondrial toxicity and hence changes in adenine nucleotide levels at high drug concentrations [29], or were reported to occur without changes in adenine nucleotides [30]. We therefore examined the effects of increasing concentrations of SBI-0206965 on the phosphorylation of Thr172 on AMPK-α, and downstream targets for AMPK, in U2OS cells (Figure 4). As expected, SBI-0206965 inhibited the MK-8722-stimulated phosphorylation of ACC (IC_50_ = 42 µM, CI 24–73), GBF1 (IC_50_ = 12 µM, CI 6–15), and Raptor (IC_50_ = 9.2 µM, CI 8–18). These values are around two orders of magnitude higher than those for BAY-3827, in keeping with the higher potency of the latter against AMPK in cell-free assays. Since SBI-0206965 is also a potent ULK1 inhibitor, we also tested its effects on the phosphorylation of Ser29 on ATG14, an established target for ULK1 [38,39]. As expected, ATG14 phosphorylation was inhibited (IC_50_ = 67 µM, CI 29–162) (Figure 4A). As observed with BAY-3827 (Figure 3), SBI-0206965 also increased Thr172 phosphorylation, but it appeared to achieve this in a biphasic manner, with a modest effect at low concentrations (3–30 µM, where the maximal effect was about 2-fold) and a larger effect at high concentrations (100–300 µM), where the effect reached 4-fold but was clearly not saturated (Figure 4A,C). Correlating with this increase in Thr172 phosphorylation, the AMPK activity measured in anti-α1/-α2 immunoprecipitates also increased with a similar biphasic pattern (Figure 4D). As a positive control, we starved the cells of all defined carbon sources in the medium (glucose, glutamine, and pyruvate) for 2 h, when the kinase activity increased almost as much as with a 30 min treatment with 300 µM of SBI-0206965 (Figure 4D). Finally, there was a very large increase (>30-fold) in the cellular AMP:ATP ratio caused by 1 h incubation with the highest concentration of SBI-0206965 (300 µM), which was even bigger than the 5-fold increase caused by the removal of glucose, glutamine, and pyruvate from the medium for 2 h.

### 2.5. Effects of Inhibitors on Inactivation by Protein Phosphatase and Activation by LKB1 in Cell-Free Assays

Thus, BAY-3827 or SBI-0206965 both promote net Thr172 phosphorylation in intact cells, but do they affect the dephosphorylation or phosphorylation of Thr172 in reconstituted cell-free assays? To study this, we immunoprecipitated endogenous AMPK from HEK-293 cells using a mixture of anti-α1 and -α2 antibodies and, following resuspension, examined the effects of the inhibitors on the rate of inactivation by PP2Cα (PPM1A) or (after dephosphorylation using PPM1A) the rate of reactivation by LKB1. This approach had the technical advantage that, because AMPK was bound to antibodies, it was easy to wash away the inhibitors and/or added phosphatase, which would otherwise have interfered with the subsequent stages of the assays. In addition, in our experience, determining the state of Thr172 phosphorylation using a kinase assay is much more quantitative than achieving this through Western blotting.

**Figure 5:**
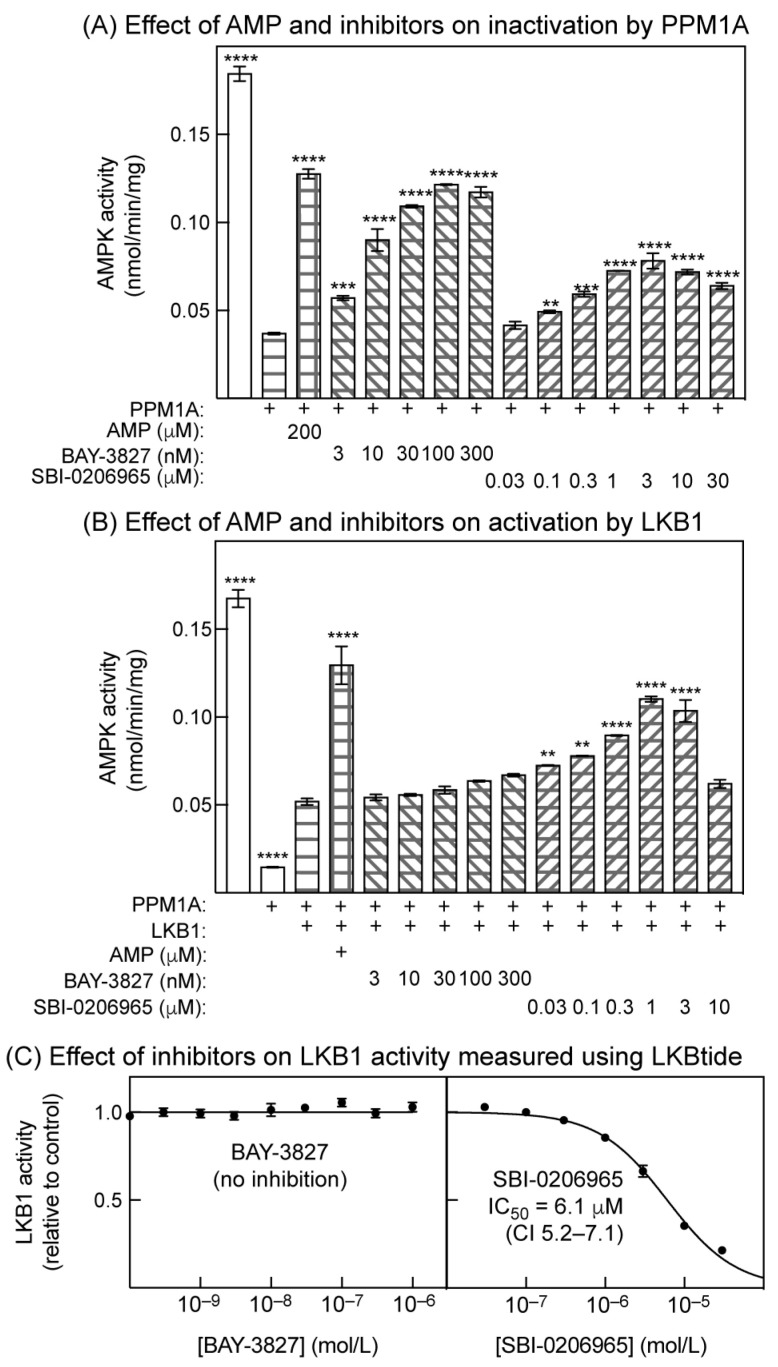
Effect of BAY-3827 and SBI-0206965 on: (**A**) inactivation by protein phosphatase-2Cα (PPM1A), and: (**B**) activation by LKB1 in cell-free assays. In both cases, endogenous AMPK was first purified from HEK-293 cells using immunoprecipitation with anti-α1 and -α2 antibodies. (**A**) AMPK was incubated in resuspended immunoprecipitates with PPM1A and Mg^2+^ in the presence or absence of AMP or the indicated concentrations of BAY-3827 or SBI-0206965. Results are mean ± SD (*n* = 2). (**B**) AMPK was incubated with PPM1A to cause Thr172 dephosphorylation and inactivation (second column). PPM1A was then removed by washing the precipitates, and the precipitates were incubated with MgATP and a fixed concentration of LKB1 (human LKB1:STRADα:MO25α complex), and various concentrations of BAY-3827 or SBI-0206965. The precipitates were washed again and assayed for AMPK activity. In (**A**), asterisks signify mean values that are significantly different from the control without AMP or AMPK inhibitor (column 2). In (**B**), asterisks signify mean values that are significantly different from the control without AMP or AMPK inhibitor (column 3). (**C**) Effect of the indicated concentrations of BAY-3827 (**left**) or SBI-0206965 (**right**) on the activity of LKB1 (LKB1:STRADα:MO25α complex) measured via phosphorylation of the LKBtide peptide. Results (mean ± SD, *n* = 2) were fitted to the equation Y = 1 − (X)/(IC_50_ + X), where IC_50_ is the concentration of SBI-0206965 causing half-maximal inhibition. The continuous curves were obtained by plotting this equation using the best-fit values for IC_50_ shown on the graph.

As expected, 200 µM of AMP provided substantial protection against the inactivation of immunoprecipitated AMPK by PPM1A (Figure 5A). Incubation with increasing concentrations of BAY-3827 or SBI-0206965 also caused progressively greater protection against inactivation by PPM1A, although the protective effect of SBI-0206965 appeared to be partially reversed at concentrations above 3 µM. Replotting of the results for BAY-3827 and SBI-0206965 showed that the half-maximal effects on protection against inactivation were at 7.0 nM (CI 4.8–10 nM) and 290 nM (CI 189–450 nM), respectively. At the concentrations used in Figure 5A, neither BAY-3827 nor SBI-0206965 inhibited PPM1A activity when measured using an alternative model substrate, para-nitrophenyl phosphate (Figure S4). The effects of the inhibitors to reduce Thr172 dephosphorylation were therefore not due to the direct inhibition of PPM1A.

Next, we immunoprecipitated AMPK from HEK-293 cells, incubated with PPM1A to cause dephosphorylation and inactivation, washed the precipitates to remove the phosphatase, incubated with a fixed concentration of ATP and LKB1 (human LKB1:STRADα:MO25α complex) in the presence or absence of the inhibitors, and measured the effects of these treatments on kinase activity (Figure 5B). As expected, 200 µM of AMP promoted activation by LKB1 by >2-fold. Increasing concentrations of SBI-0206965 also progressively promoted activation by LKB1, although the effect was reversed at concentrations above 1 µM. However, BAY-3827 had little or no effect on activation by LKB1—while there appeared to be a trend towards the promotion of activation, this was not statistically significant at any concentration of BAY-3827 used in Figure 5B.

We suspected that the reversal of the effect of SBI-0206965 to promote Thr172 phosphorylation by LKB1 at higher concentrations might have been due to the inhibition of the latter kinase by the inhibitor. Although it was reported that SBI-0206965 did not inhibit LKB1 [29], those assays were carried out at a single fixed inhibitor concentration (0.25 µM). We assayed the effects of different concentrations of BAY-3827 and SBI-0206965 on LKB1 activity (Figure 5C) measured using LKBtide, a peptide substrate based on the sequence around the LKB1 site on the kinase NUAK2. While BAY-3827 had no effect on LKB1 activity at any concentration used in Figure 5B, SBI-0206965 caused the progressive inhibition of LKB1 activity at concentrations of 1 µM and above (IC_50_ = 6.1 µM). This is the most likely explanation for the reversal of the stimulatory effects of SBI-0206965 on Thr172 phosphorylation by LKB1 at concentrations above 1 µM (Figure 5B).

### 2.6. BAY-3827 Bio-Availability Is Low following Oral Administration in Mice

**Figure 6:**
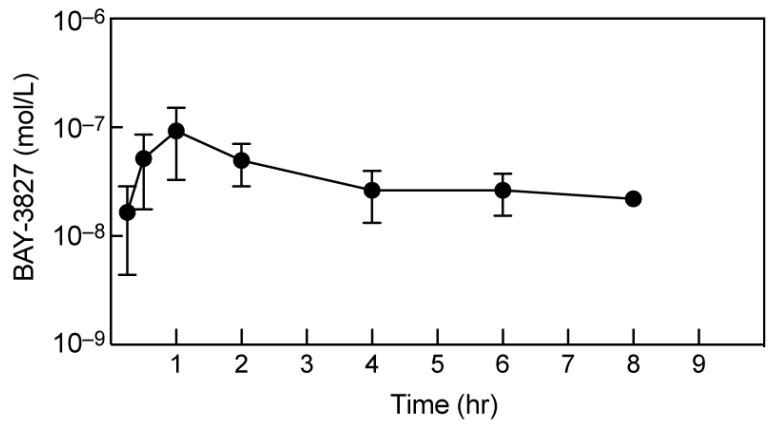
Recovery of BAY-3827 in the bloodstream of NOD-SCID mice at various times after administration of the inhibitor (10 mg/kg) via oral gavage. The cytochrome P450 inhibitor 1-aminobenzotriazole (50 mg/kg) had been administered via oral gavage 30 min prior to BAY-3827 in an attempt to improve oral bioavailability. The inhibitor was measured using LC:MS in blood samples taken at the indicated time after administration. Results are mean ± SD (*n* = 2); note the logarithmic scale for inhibitor concentration.

Given the very effective inhibition of AMPK by BAY-3827 in cellular studies, and the current lack of availability of selective AMPK inhibitors, we hoped to use this compound to study the functions of AMPK in vivo. We therefore performed pilot experiments to gauge the doses and routes of administration required. Unfortunately, the aqueous solubility of the compound was low (0.3 µg/mL), consistent with the data available on the Structural Genomics Consortium website [32]. In the same source, the in vitro clearance by rat hepatocytes (CL = 3.3 L/h/kg) or mouse liver microsomes (CL = 5 L/h/kg) was also reported to be high, and the oral bio-availability was reported to be low (Fmax = 21% for rat hepatocytes and 7% for mouse liver microsomes), which was confirmed by in-house analyses. In an attempt to improve the oral availability, we co-administered BAY-3827 (10 mg/kg) via oral gavage with 1-aminobenzotriazole (50 mg/kg), a general inhibitor of the cytochrome P450s, that modifies most hydrophobic drugs [40]. BAY-3827 peaked in the blood at a mean of 90 nM at 60 min, and then declined to a mean of 20 nM after 8 h (Figure 6). Since the IC_50_ for the inhibition of ACC phosphorylation in U2OS cells was 270 nM (Figure 3), with similar results in two other human cell lines (HEK-293 and NCI-H2009, not shown), BAY-3827 is unlikely to be efficacious after administration via this route.

## 3. Discussion

We confirmed that BAY-3827 is an extremely potent inhibitor of AMPK in cell-free assays. Under standard assay conditions (ATP concentration: 200 µM), BAY-3827 was >20-fold more potent against purified rat liver AMPK than SBI-0206965 (IC_50_ values 17 nM and 360 nM, respectively). BAY-3827 was also an order of magnitude more potent than SBI-0206965 against the bacterially expressed human α1β1γ1 and α2β2γ1 complexes, with the IC_50_ for SBI-0206965 we obtained for α1β1γ1 (with ATP at 200 µM) being 250 nM, comparable with the previous estimate of 300 nM by Dite et al. [29].

BAY-3827 was also a more potent inhibitor than SBI-0206965, by around two orders of magnitude, when tested against the phosphorylation of AMPK targets (ACC, Raptor, and GBF1) in intact U2OS cells. It is therefore the preferred choice as an AMPK inhibitor in cellular studies, although it does inhibit a small number of other kinases with a similar potency to AMPK, including all four isoforms of p90 ribosomal protein S6 kinase (RSK1-4, also known as RPS6KA1-4) (Figure S1C). Since the off-target effects of BAY-3827 and SBI-0206965 against other protein kinases do not show a significant overlap, there may also be a case for using both inhibitors side-by-side.

The IC_50_ value for BAY-3827 we obtained with the isolated α2 kinase domain (89 nM) was very similar to that obtained for the α2β2γ1 holoenzyme (70 nM), suggesting that the inhibitor primarily inhibits AMPK by binding to the kinase domain on the α subunit. BAY-3827 behaved as a mixed (competitive and uncompetitive) inhibitor relative to the substrate MgATP. This is similar to previous results with SBI-0206965 [29] and suggests that the binding sites for MgATP and BAY-3827 overlap, while not being identical. The structure of the human α2 kinase domain (residues 6–278, phosphomimetic T172D mutant) in complex with SBI-0206965 was solved using X-ray crystallography [29], and this confirmed that the binding sites for MgATP and SBI-0206965 overlap. Although BAY-3827 and SBI-0206965 are not closely related structurally, both contain a benzamide moiety that is found in many Type II kinase inhibitors (Figure S2). This moiety often displaces the DFG motif at the start of the activation loop into the “DFG-out” rather than “DFG-in” conformation, in which the aspartate side chain within the motif is no longer correctly positioned to bind the Mg^2+^ ion of the substrate, Mg.ATP^2−^ [34]. Although there is no structure available for an AMPK kinase domain with bound BAY-3827, a diagram on the Structural Genomics Consortium website [32] shows part of a structure of the human α2 kinase domain in complex with the closely related lead compound, compound 6 (Figure S2), an AMPK inhibitor with an IC_50_ just 10-fold higher than BAY-3827. This reveals that one side of the benzamide moiety of compound 6 forms hydrogen bonds with the hinge region of the ATP-binding site, while the other side interacts with Phe169, which lies on the activation loop very close to Thr172. Moreover, the dihydropyridine ring of the inhibitor is enclosed within a pocket “formed by activation loop induced fit” [32]. Thus, while BAY-3827 is likely to block the ATP-binding site, it also seems likely that its binding alters the conformation of the activation loop.

Our results show that the binding of BAY-3827 interacts with allosteric activation by both AMP and MK-8722, because the concentration of these agents causing half-maximal activation (EC_50_) increased by 5- and 7-fold, respectively, in the presence of BAY-3827. Since the critical sites for the binding of AMP (the CBS3 site on the γ subunit) and MK-8722 (the ADaM site) are located at some distance from each other [18,41], these effects are unlikely to be due to direct steric hindrance by the binding of BAY-3827. It is more likely that the conformational change induced by the binding of the inhibitor to the kinase domain restricts the conformational changes induced by the two allosteric activators. In a similar vein, the phosphorylation of Thr172 in the α1β1γ1 complex greatly reduces maximal activation by the ADaM site activator A-769662 [42], despite the fact that Thr172 and the ADaM site are some distance apart in existing structures [18,41].

We also show here that both BAY-3827 and SBI-0206965 completely inhibit the phosphorylation in intact U2OS cells of three established downstream targets for AMPK that are located in different subcellular compartments, i.e., Ser80 on ACC (cytoplasm), Ser792 on Raptor (lysosome), and Thr1337 on GBF1 (Golgi apparatus). However, inhibition by BAY-3827 was around two orders of magnitude more potent than that by SBI-0206965, suggesting once again that BAY-3827 should be the inhibitor of choice for cellular studies. This view was reinforced by the finding that the phosphorylation of Ser29 on ATG14, known to be downstream of the alternate target of SBI-0206965, ULK1, was also inhibited by SBI-0206965 at similar concentrations to the AMPK target sites. An additional disadvantage of SBI-0206965 is that, at concentrations required to give a complete blockade of the phosphorylation of AMPK targets (300 µM), the compound increased cellular AMP:ATP ratios and activated AMPK via the canonical mechanism. Other authors [29,30] may have avoided this complication by using non-saturating concentrations of SBI-0206965 below 100 µM, although a small decrease in adenylate energy charge (albeit not statistically significant) was observed in HEK-293 cells treated with 30 µM of SBI-0206965 for 1 h [29]. Although we did not establish the mechanism through which SBI-0206965 increased AMP:ATP ratios, a likely explanation is that it inhibits mitochondrial oxidative phosphorylation, similar to many AMPK activators [43]. Another drawback of SBI-0206965 is that, at concentrations of 3 µM and above, the promotion of AMPK activation by LKB1 in cell-free assays was reversed (Figure 5B), almost certainly because SBI-0206965 inhibited LKB1 activity at these concentrations, shown when measured independently using the substrate peptide, LKBtide (Figure 5C). By contrast, BAY-3827 did not have these off-target effects at any concentration used to inhibit AMPK in this paper.

The most unexpected findings of this study were that both BAY-3827 and SBI-0206965, while blocking the phosphorylation of downstream targets of AMPK as expected, at similar concentrations paradoxically increased the phosphorylation of AMPK at Thr172. This would be expected to cause an anomalous activation of AMPK, and indeed, this could be measured in immunoprecipitates prepared from cells treated with these compounds. Since Thr172 phosphorylation is often used as a surrogate marker for AMPK activation, it is important that researchers using these compounds as AMPK inhibitors should be aware of this paradox.

How do these inhibitors cause increases in net Thr172 phosphorylation in intact cells? One possibility is that they cause increases in cellular AMP:ATP ratios, which would increase net Thr172 phosphorylation through the well-established, canonical mechanisms. However, in the case of BAY-3827, no increases in AMP:ATP ratio were observed at any concentration used in the intact cells. In the case of SBI-0206965, higher concentrations (300 µM) did cause increases in cellular AMP:ATP ratios, correlating with the more extensive Thr172 phosphorylation seen at these concentrations, but there were also significant increases in Thr172 phosphorylation at lower concentrations (3–100 µM), where increases in AMP:ATP ratios were not observed. A structural analysis suggests that SBI-0206965 is a Type II inhibitor whose binding prevents the “DFG-in” conformation [29], while we have argued above, based on the structure of the α2 kinase domain with compound 6 [32], that the binding of BAY-3827 is also likely to affect the conformation of the activation loop. These conformational changes in the activation loop may then either expose Thr172 for phosphorylation or protect it against dephosphorylation. Surprisingly, our cell-free assays in Figure 5 suggest that BAY-3827 and SBI-0206965 promote net Thr172 phosphorylation through somewhat different mechanisms. Thus, the binding of BAY-3827 clearly protected against inactivation by the protein phosphatase PPM1A, while having much more modest effects, if any, on phosphorylation and activation by the LKB1 complex. Conversely, at low concentrations (0.1–1 µM), SBI-0206965 appears to modestly promote phosphorylation and activation by the LKB1 complex, while also protecting against dephosphorylation and inactivation by PPM1A. At higher concentrations (>3 µM), both of these effects appeared to be partially reversed in the cell-free assays, but in the intact cells, the net phosphorylation of Thr172 continued to increase at higher concentrations of SBI-0206965, most likely because the inhibitor increases cellular AMP:ATP ratios, with the consequent effects of elevated AMP on the phosphorylation and dephosphorylation of Thr172.

We recently reported that SU6656, although originally developed as an inhibitor of Src family tyrosine kinases, is also an active site-directed inhibitor of AMPK that is competitive with ATP (Ki = 170 nM), which, like BAY-3827 and SBI-0206965, promotes Thr172 phosphorylation in intact cells [44]. However, a major difference was that, in the case of SU6656, this was associated with the activation of AMPK in the intact cells, as assessed by the increased phosphorylation of ACC. We argued [44] that this anomalous behaviour could be explained by assuming that SU6656 binding caused a conformational change in the activation loop that promoted Thr172 phosphorylation, and that the lifetime of the active, phosphorylated state was sufficiently long to allow for the dissociation of the inhibitor and its replacement by ATP, so that one or more cycles of catalysis could occur before Thr172 was dephosphorylated. In the case of BAY-3827 and SBI-0206965, the re-association of the inhibitor may be much more rapid compared to the binding of ATP, so that that the overall effect observed is a decrease rather than an increase in the phosphorylation of downstream targets.

Interestingly, in budding yeast (*Saccharomyces cerevisiae*) carrying a mutation (I132G) that renders Snf1 (yeast ortholog of AMPK-α1 and -α2) sensitive to inhibition by the ATP analog 2-naphthylmethyl pyrazolopyrimidine-1, the addition of the inhibitor caused increased phosphorylation of Thr210 (equivalent to mammalian Thr172) [45,46]. This paradoxical effect of kinase inhibitors may therefore be a general feature of AMPK orthologs from diverse species.

Unfortunately, our pharmacokinetic analyses of BAY-3827, together with previous results summarized on the Structural Genomics Consortium website [32], suggest that it is rapidly metabolized by the liver, such that its metabolic stability in vivo is low. Even when combined with a cytochrome P450 inhibitor in our pharmacokinetic study (Figure 6), its peak level in the bloodstream (≈90 nM) at 1 h after oral administration was only one third of the IC_50_ for the inhibition of ACC phosphorylation in U2OS cells (270 nM), and its level then declined to 20–30 nM after 4–8 h. We considered other routes of administration such as intraperitoneal injection, but these were ruled out by the low solubility of BAY-3827. Thus, further chemical modification of the compound may be necessary before it is a useful reagent for use in vivo. SBI-0206965 also has a short elimination half-life and low relative oral availability [47].

In conclusion, BAY-3827 is to be much preferred over both compound C and SBI-0206965 as an AMPK inhibitor for cellular studies on AMPK function. It is more selective for AMPK than SBI-0206965 and considerably more selective than compound C. Another disadvantage of SBI-0206965 is that, at the concentrations required to achieve the complete inhibition of the phosphorylation of AMPK downstream targets (100 and 300 µM), it disturbs the cellular energy status. By contrast, BAY-3827 is about two orders of magnitude more potent as an AMPK inhibitor in intact cells and, at the concentrations required to completely inhibit the phosphorylation of downstream AMPK targets, does not disrupt the cellular energy status. We therefore recommend that, if a single inhibitor is used, BAY-3827 should be used instead of SBI-0206965 or compound C in future studies of AMPK function utilizing cultured cells, although it may be less suitable for use in vivo. It may, however, be necessary to establish that any effects observed are not due to the inhibition of the very small number of other kinases (e.g., RSK1-4) that are also potently inhibited by BAY-3827.

## 4. Materials and Methods

### 4.1. Cell Culture

T-Rex U2OS cells carrying a Flp Recombinase Target (FRT) site were generated as described previously [48] and grown in Dulbecco’s Modified Eagle’s Medium (DMEM) supplemented with 10% (*v*/*v*) FBS and 1% (*v*/*v*) penicillin/streptomycin.

### 4.2. Antibodies and Other Probes

Phosphospecific antibodies were as described: pT172 (AMPK-α: Cell Signaling Technology, Danvers, MA, USA, Cat# 2535, RRID AB_331250), pS79/pS212 (ACC1/ACC2: Cell Signaling Technology, Cat# 11818, RRID AB_2687505), pS792 (Raptor: Cell Signaling Technology, Cat# 2089146, RRID AB_2934061), pT1337 (GBF1: IBL International, Hamburg, Germany; Cat# 28065-IBL, RRID AB_2232232), and pS29 (ATG14: Cell Signaling Technology, Cat# 92340, RRID AB_2800182). Antibodies against total proteins were as described: AMPK-α1 and AMPK-α2 (for immunoprecipitation): [49]; AMPK-α (pan-α, for Western blotting): Cell Signaling Technology, Cat#2793, RRID AB_915794GBF1; Raptor: Cell Signaling Technology, Cat# 2280, RRID AB_561245; GBF1: BD Biosciences, San Jose, CA, USA, Cat# 28065-IBL, RRID AB_2232232; ATG14: MBL International, Woburn, MA, USA, Cat# PD026, RRID AB_1953054; tubulin: Sigma-Aldrich, St. Louis, MO, USA, Cat# T4026, RRID AB_477577; α-actin: Sigma-Aldrich, Cat# A2228, RRID AB_476697. Streptavidin conjugated to 800 nm fluorophore (for the detection of total ACC) was from Rockland Immunochemicals, Pottstown, PA, USA, Cat# S000-32.

### 4.3. Chemicals, Peptides, and Recombinant Proteins

LKBtide, Cat# 12-540, donepezil hydrochloride, ≥98%, Cat# D6281; phenformin hydrochloride, Cat# P7045; and berberine chloride, Cat# B3251, were from Sigma-Aldrich; p-nitrophenyl phosphate was from New England Biolabs, Ipswich, MA, USA, Cat# PO757S. BAY-3827 and BAY974 were from the Structural Genomics Consortium, Toronto, ON, Canada, (donated chemical probes); MK-8722 was synthesized in-house, as in [50]. SBI-0206965 was from Selleckchem, Houston, TX, USA, Cat# S7885. Acetonitrile (LC:MS grade), Cat# A19862.AP and formic acid (LC:MS grade), Cat# 270480250) were from ThermoFisher Scientific, Waltham, MA, USA. AMPK was purified from rat liver, as in [8]. Human (His)_6_-tagged AMPK (α1β2γ1, bacterially expressed, generated as in [51], was a gift from Dr Mark Peggie, University of Dundee, Dundee, UK; human (His)_6_-tagged AMPK (α2β2γ1, bacterially expressed) generated as in [51] was a gift from AstraZeneca, Cambridge, UK; human GST-tagged α2 kinase domain (1–310) was produced as described [44]; and human LKB1:STRAD-α:MO25-α complex [52], GST-tagged CaMKK2 [53], and PP2Cα (PPM1A) [54] were produced as described.

### 4.4. Cell Lysis

Cells were lysed rapidly in situ on culture plates using ice-cold lysis medium containing phosphatase inhibitors, as described previously [55].

### 4.5. Kinase Assays

AMPK assays measured the transfer of radioactivity from labelled ATP to a peptide substrate, which was separated from unreacted ATP by binding to P81 paper. This assay was described previously [56], except that we used [γ-^33^P]ATP rather than [γ-^32^P]ATP. The peptide substrate was the *AMARA* peptide in all experiments other than one, which analyzed allosteric activation by AMP (Figure 2A), when, for technical reasons, the *SAMS* peptide was used instead [56]. With purified preparations of AMPK, it was assayed in solution, but for assays in cell lysates (Figure 3D and Figure 4D), it was first immunoprecipitated using a mixture of anti-α1 and -α2 antibodies and assayed in resuspended immunoprecipitates [56]. After the initial immunoprecipitation of lysate containing 80 µg of protein, the pellets were washed twice with 1 mL of high salt IP buffer (50 mM Tris-HCL, pH 7.4 at 4 °C, 1 M NaCl, 1 mM EDTA, 1 mM EGTA, 50 mM NaF, 5 mM Na pyrophosphate, 1 mM dithiothreitol, 0.1% (*v*/*v)* Triton X-100) and twice with 1 mL of low salt IP buffer (as above but only 150 mM NaCl), followed by a final wash in 1 mL of assay buffer (50 mM Na Hepes, pH7.4, 150 mM NaCl, 1 mM dithiothreitol, 0.02% (*v*/*v*) Brij-35). After each wash, the pellet was recovered via centrifugation (16,000× *g*, 3 min) and then resuspended. The final resuspended pellets from each biological replicate were divided into two aliquots (40 µg lysate protein) to provide technical replicates and assayed as above.

### 4.6. Phosphorylation and Activation of Bacterially Expressed AMPK

Bacterially expressed heterotrimers and kinase domain constructs (Figure 1B–D) were activated through Thr172 phosphorylation using ATP and GST-tagged CaMKK2, and CaMKK2 was removed via passage through glutathione-Sepharose, as described previously [57].

### 4.7. Assays of AMPK Dephosphorylation by PP2Cα (PPM1A)

AMPK was immunoprecipitated from HEK-293 cell lysate (2.5 mg of lysate protein) using a mixture of anti-α1 and -α2 antibodies. After extensive washing as in Section 4.5, aliquots equivalent to 80 µg of lysate protein were incubated in a shaking incubator at 30 °C for 10 min in Hepes buffer (50 mM Na Hepes, pH 7.4, 150 mM NaCl, 1 mM dithiothreitol, 0.02% (*w*/*v*) Brij-35) with 50 mM MgCl_2_ and sufficient PPM1A to yield about 80% inactivation, in the presence or absence of 200 µM AMP or the indicated concentration of BAY-3827 or SBI-0206965 (total volume 50 µL). The reaction was terminated by adding 1 mL of IP buffer (50 mM Tris/HCl, pH 7.4 at room temperature, 150 mM NaCl, 1 mM EDTA, 1 mM EGTA, 5 mM Na pyrophosphate, 50 mM NaF, 1 mM dithiothreitol, 0.1% (*v*/*v*) Triton X-100) and, after additional washing in the same buffer and a final wash into Hepes buffer, AMPK activity was determined using kinase assays as above.

### 4.8. Assays of AMPK Phosphorylation by the LKB1 Complex

AMPK was immunoprecipitated from HEK-293 cell lysate (2.5 mg protein) using a mixture of anti-α1 and -α2 antibodies. After extensive washing, it was incubated in a shaking incubator at 30 °C for 20 min in Hepes buffer with 50 mM MgCl_2_ and sufficient PPM1A to yield about 90% inactivation (total volume of 250 µL). The reaction was terminated by adding 1 mL of IP buffer and, after additional washing in the same buffer, aliquots equivalent to 80 µg of lysate protein were incubated in a shaking incubator at 30 °C for 10 min in Hepes buffer with 200 mM ATP and 5 mM MgCl_2_, with or without a limiting amount of LKB1:STRADα:MO25α complex (0.5 µg) in the presence or absence of 200 µM AMP or the indicated concentration of BAY-3827 or SBI-0206965 (total volume of 50 µL). The reaction was terminated by adding 1 mL of IP buffer and, after additional washing in the same buffer and a final wash into Hepes buffer, AMPK activity was determined using kinase assays as above.

### 4.9. Assays of LKB1 Using LKBtide and PPM1A Using P-Nitrophenyl Phosphate

To directly determine LKB1 activity, kinase assays were performed in the presence or absence of the indicated concentration of BAY-3827 or SBI-0206965 as for AMPK, except that LKBtide was used as a peptide substrate. PPM1A activity was determined using p-nitrophenyl phosphate (pNPP) as a substrate. PPM1A (5 µg) was incubated with 50 mM pNPP and 50 mM MgCl_2_ in Hepes buffer in the presence of a vehicle or the indicated concentration of BAY-3827 or SBI-0206965 for 10 min at 30 °C. The reaction was quenched by the addition of 1 mL of 0.5 M EDTA and the amount of product, p-nitrophenol, determined by measuring absorbance at 405 nm.

### 4.10. Pharmacokinetic Study in Mice

All experiments with live animals were approved in advance by the Ethical Review Committee of the University of Dundee in accordance with the UK Animal (Scientific Procedures) Act 1986. Two female mice of the NOD-SCID strain were obtained from Charles River Laboratories, UK. The animals were maintained under a 12 h light/12 h dark cycle. The animals were allowed ad libitum access to food (RM1; Special Diet Services, UK) and water. Temperature and relative humidity were maintained between 20 and 24 °C and 45 and 65%, respectively. All animals received a minimum of 14 days acclimatization prior to the start of the study. 1-Aminobenzotriazole was dissolved on the day of dosing in Milli-Q ultrapure water at a concentration of 10 mg/mL and dosed via oral gavage at a dose level of 50 mg/kg. BAY-3827 was suspended in 0.5% hydroxypropylmethylcellulose (Methocel K15M Premium USP/EP; Colorcon) in water, at a concentration of 2 mg/mL. The BAY-3827 formulation was dosed via oral gavage at a dose level of 10 mg/kg, 30 min after the administration of 1-aminobenzotriazole. The animals were observed regularly after dose administration. Serial blood samples (10 µL per sample) were collected from the lateral tail vein prior to dosing, then at 5, 15, and 30 min, and 1, 2, 4, 6, and 8 h after the administration of BAY-3827. Each blood sample was diluted into 90 µL of Milli-Q ultrapure water and stored at −20 °C prior to analysis.

### 4.11. Analysis of Blood Levels of BAY-3827

A Waters Acquity Performance Liquid Chromatography coupled to a Waters Xevo TQ-S tandem mass spectrometer was used for the quantification of BAY-3827, using donepezil hydrochloride as an internal standard. System control, data acquisition, and data processing were performed using MassLynx^®^ (Version 4.2). Chromatography was performed on an Acquity UPLC BEH C18 column, 50 × 2.1 mm, particle size 1.7 µm (Waters Part No.: 186002350). The column was kept at 40 °C and prepared samples were maintained at 4 °C in the sample manager. Mobile phase A was LC:MS grade water containing 0.01% (*v*/*v*) formic acid, and mobile phase B was acetonitrile containing 0.01% (*v*/*v*) formic acid. The gradient was run at a flow rate of 0.6 mL/min, starting at 95% A and 5% B. After 2 min, the gradient was changed to 5% A and 95% B and kept stable until 2.5 min. At 2.6 min, the gradient was changed back to the starting conditions and held until 3.4 min. The sample injection volume was 1 µL. The tandem mass spectrometer was operated in positive mode electron spray ionisation (ESI) with multiple reaction monitoring acquisition parameters shown in Table 2. The source and desolvation temp. were set at 150 °C and 600 °C, respectively. Nitrogen was used as the desolvation gas and flow was set at 1000 L/h.

### 4.12. Preparation of Internal Standards

Internal standard stock solution was prepared in DMSO to obtain a concentration of 1 mg/mL of donepezil hydrochloride. A working solution of IS was prepared in acetonitrile to obtain a final concentration of 50 ng/mL.

### 4.13. Preparation of BAY-3827 Solutions

Two individual BAY-3827 stock solutions were prepared in DMSO to obtain a concentration of 1 mg/mL for the creation of the calibration and quality control (QC) working solutions. The BAY-3827 calibration working solutions were prepared in acetonitrile:water (50:50 *v*/*v*) spanning the range from 0.002 to 40 µg/mL. The serial calibration concentrations of BAY-3827 were prepared in control mouse blood at concentrations of 0.1, 0.2, 0.4, 1, 2, 4, 10, 20, 40, 100, 200, 400, 1000, and 2000 ng/mL. The concentrations of quality control (QC) samples, in the control mouse blood, were prepared at 1, 4, 20, 100, and 400 ng/mL.

### 4.14. Sample Preparation

Calibration samples, QC samples, blanks, double blanks, and study samples were extracted through protein precipitation. A volume of acetonitrile containing internal standard (300 µL) was added to all calibration samples, QC samples, and blanks (100 µL). In total, 300 µL of acetonitrile alone (no IS) was added to all double blank samples (100 µL). For the study samples (30 µL), 90 µL of acetonitrile containing internal standard was added. All samples were then vortex mixed for 30 s and centrifuged (SIGMA SciQuip 1-14, 10 min, 14,800 rpm/16,163× *g*) to pellet the proteins. The resultant supernatant from the calibration samples, QCs, double blanks, and blanks (200 µL) and study samples (80 µL) was then transferred to HPLC glass vials containing Milli-Q water (100 µL or 40 µL, respectively) to reduce the solvent strength ready for UHPLC/MS analysis.

### 4.15. LC:MS Sample Analysis

The sample concentrations were determined via a linear regression (weighting: 1/x) on the calibration samples using Waters TargetLynx application (v4.2). Non-zero calibrators were ±20% of the nominal (theoretical) concentrations, and data points that failed to meet this acceptance criteria were excluded. The lower limit of quantification (LLoQ) was determined as the analyte response with ≥three times the analyte response of the blanks (plasma with internal standard but not analyte). For the sample extraction run, the dynamic range of the BAY-3827 calibration line was 0.2–100 ng/mL. Quality control samples were injected throughout the analytical run, with an acceptance criterion of 66% of the injected samples being within ±20% of the nominal (theoretical) concentrations. All blanks and double blanks (plasma with no internal standard or analyte) were verified as being free of interference at the retention times of the analyte and internal standard. Sample carryover was determined in a blank injection immediately following the injection of the top calibration sample. Carryover was analytically accepted when the analyte response in the blank was <1% of the analyte response in the top calibration sample.

### 4.16. Other Analytical Procedures

SDS-PAGE was performed using precast NuPAGE Tris-acetate 3–8% gradient polyacrylamide gels in Tris-acetate running buffer when analyzing ACC, GBF1, or Raptor, or NuPAGE Bis-Tris 4–12% gradient polyacrylamide gels in the MOPS buffer system (ThermoFisher Scientific, Waltham, MA, USA) for other proteins (unless stated otherwise). Proteins were transferred to nitrocellulose membranes using the iBlot 2 system (ThermoFisher Scientific). Membranes were enhanced if necessary using SuperSignal Western Blot Enhancer (ThermoFisher Scientific), then blocked for 1 h in Li-Cor Odyssey blocking buffer. The membranes were probed with the appropriate primary antibody (0.1–1 mg/mL) overnight at 4 °C. Detection was performed using secondary antibody or streptavidin probe (for total ACC) coupled to IR 680 or IR 800 dye, and the membranes were scanned using the LICOR Odyssey IR imager. Protein concentrations were determined by Coomassie Blue binding with bovine serum albumin as a standard [58]. The cellular levels of AMP and ATP were determined via direct perchloric acid extraction from cells, with LC:MS used to estimate the levels of each nucleotide [59].

### 4.17. Curve Fitting

Data were fitted to the specified equation with a non-linear regression analysis using GraphPad Prism 6 for Mac OSX.

### 4.18. Quantification and Statistical Analysis

The numbers of replicates and statistical significance are indicated in figures or figure legends. Error bars are ±Standard Deviation (SD) or ±Standard Error of the Mean (SEM), as specified in the figure legends; they were omitted if they were smaller than the symbol used for the mean value. Numbers of replicates (*n*) refer to biological replicates, i.e., the number of independent cell cultures analyzed, independent cell-free assays conducted, or mice used. The significances of these differences were estimated with GraphPad Prism 6 for Mac OSX, using 1-way or 2-way ANOVA, as appropriate, and (unless stated otherwise) the Holm–Sidak multiple comparison test. Significant differences are indicated by asterisks: * *p* < 0.05, ** *p* < 0.01, *** *p* < 0.001, and **** *p* < 0.0001, or daggers: ^†^ *p* < 0.05, ^††^ *p* < 0.01, ^†††^ *p* < 0.001, and ^††††^ *p* < 0.0001.

## Figures and Tables

**Table 1:** Apparent kinetic parameters for MgATP in the presence of increasing concentrations of BAY-3827, assayed using the bacterially expressed human α2 kinase domain after phosphorylation of Thr172 using CaMKK2. CI, 95% confidence interval.

	Control	BAY-3827 (10 nM)	BAY-3827 (30 nM)	BAY-3827 (100 nM)	BAY-3827 (300 nM)
Apparent Km (µM)	309	382	391	887	1480
CI Km (µM)	253–378	326–446	357–429	825–954	1224–1807
Apparent Vmax (µmol/min/mg)	2.19	2.09	1.78	1.69	1.27
CI Vmax (µmol/min/mg)	2.07–2.32	2.00–2.20	1.73–1.83	1.65–1.74	1.22–1.81

**Table 2:** LC:MS Acquisition Parameters.

Compound	Parent (*m*/*z*)	Daughter (*m*/*z*)	Cone Voltage (V)	Collision Energy (V)
BAY-3827	469.2	132.6	12	30
Donepezil	380.2	90.9	60	33

## Data Availability

Excluding pilot studies, all data generated during this project are included in the manuscript.

## Supplementary Materials

The supporting information can be downloaded at: https://www.mdpi.com/article/10.3390/ijms25010453/s1.
