# Supplementary material for: BAY-3827 and SBI-0206965: Potent AMPK Inhibitors That Paradoxically Increase Thr172 Phosphorylation
Source: Int J Mol Sci. 2023 Dec 29;25(1):453. doi: 10.3390/ijms25010453 (PMC10778976; doi:10.3390/ijms25010453)

Figure S1

A) Heat maps summarizing relative selectivity of AMPK inhibitors

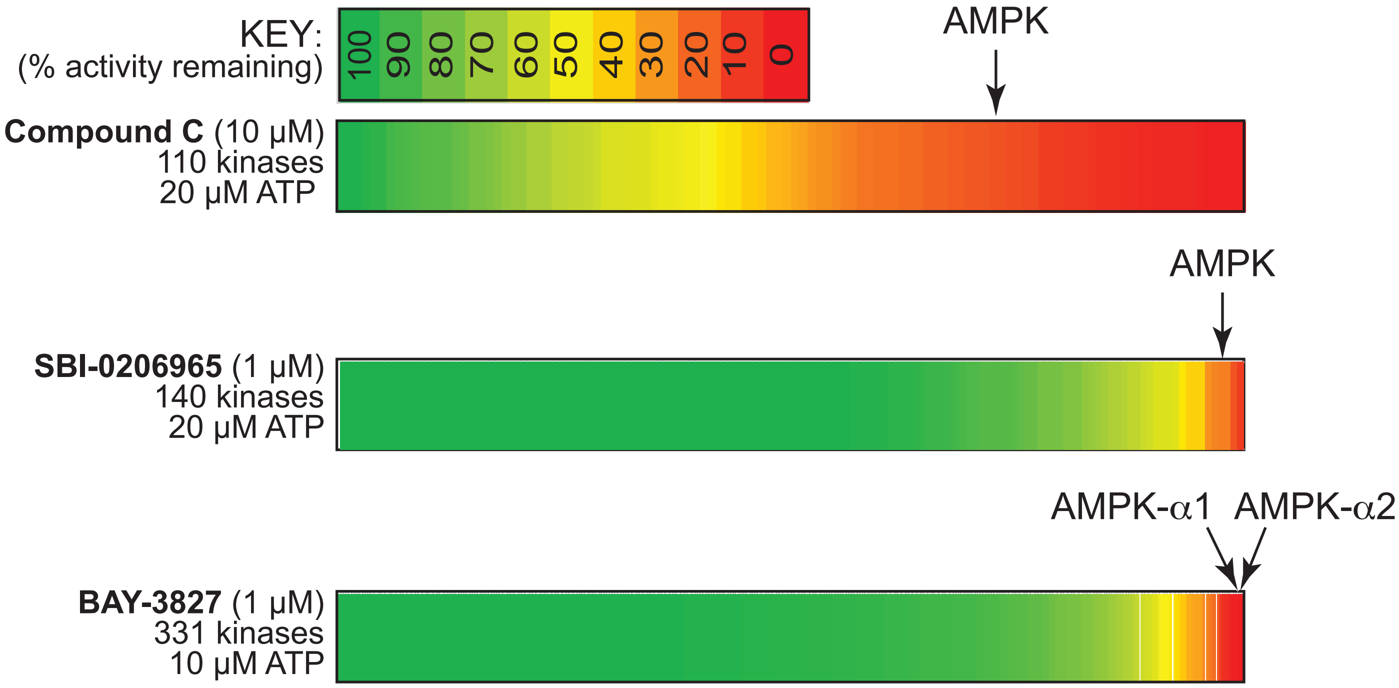

B) inhibition of protein kinases by SBI-0206965 (top tenth percentile only)

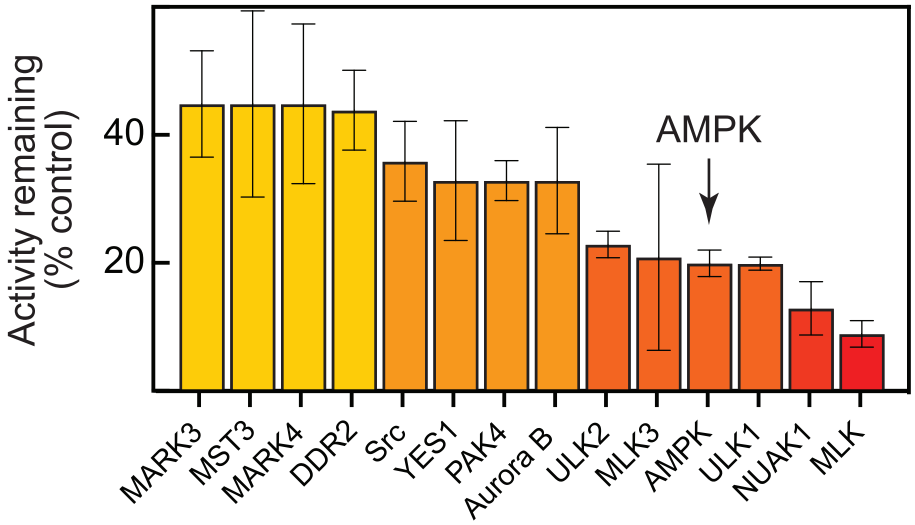

C) inhibition of protein kinases by BAY-3827 (top tenth percentile only)

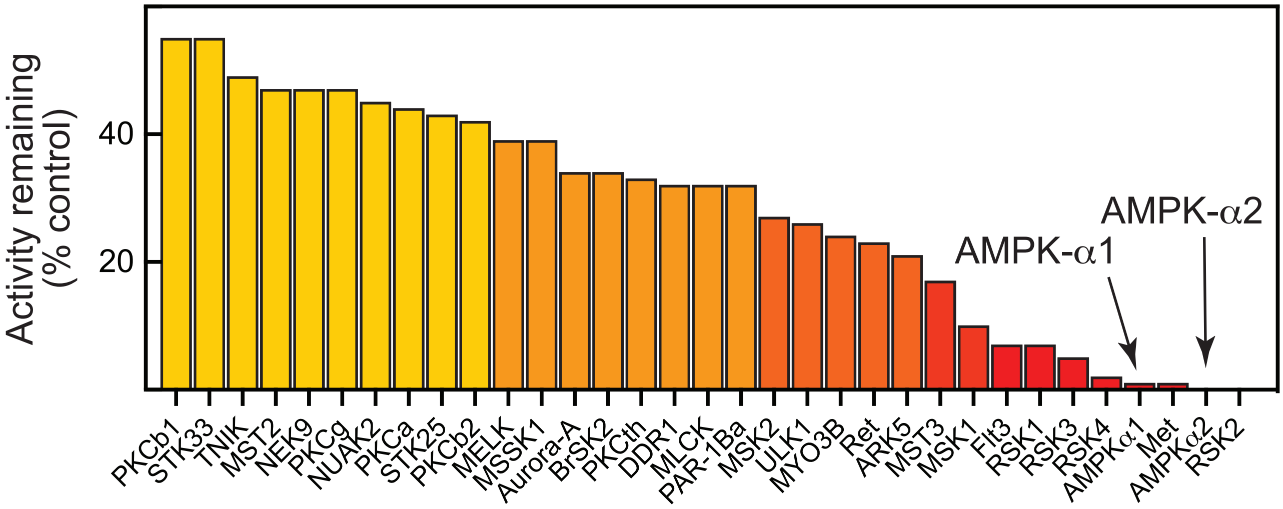

Supplement: Supplementary file 1 [file ijms-25-00453-s001.zip › FigS1.pdf]
