# Supplementary material for: BAY-3827 and SBI-0206965: Potent AMPK Inhibitors That Paradoxically Increase Thr172 Phosphorylation
Source: Int J Mol Sci. 2023 Dec 29;25(1):453. doi: 10.3390/ijms25010453 (PMC10778976; doi:10.3390/ijms25010453)
Supplement: Supplementary file 1 [file ijms-25-00453-s001.zip › FigS3.pdf]

Figure S3

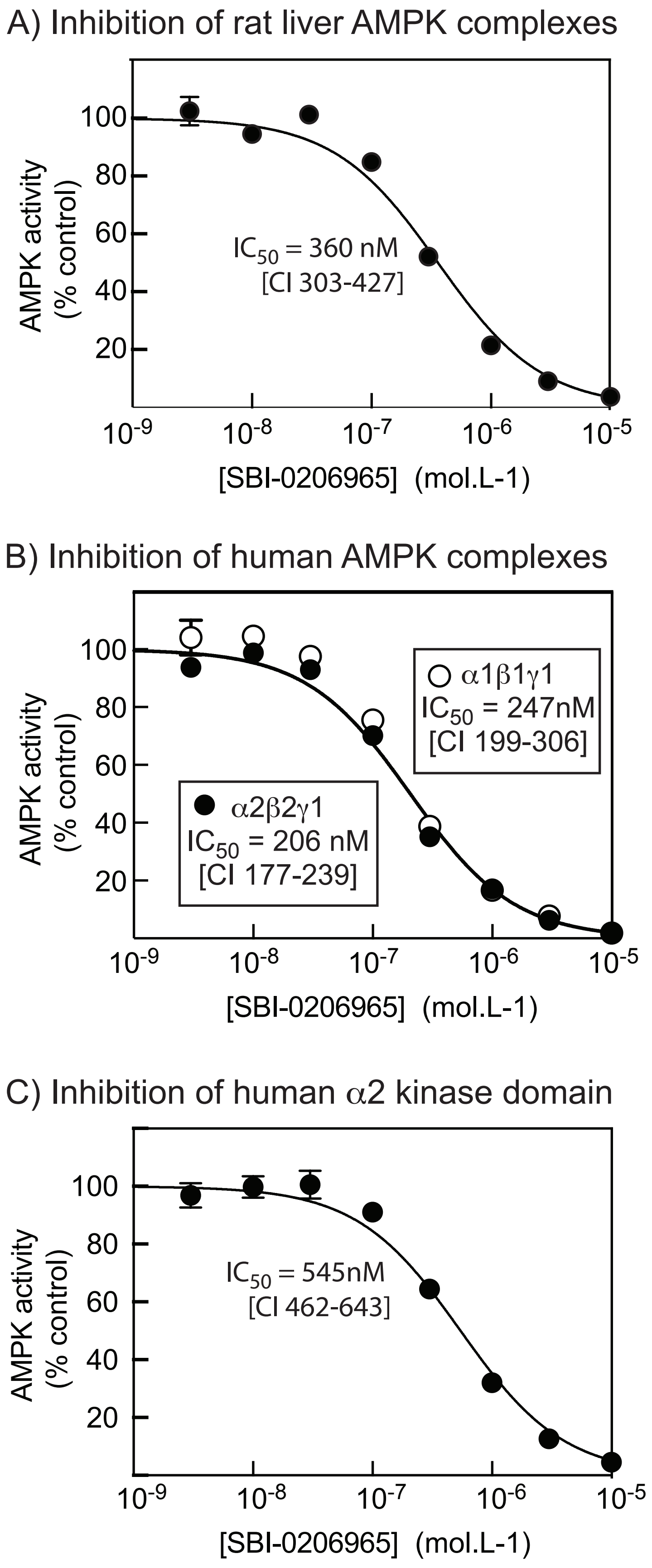
A) Inhibition of rat liver AMPK complexes
AMPK activity (% control)
100
80
60
40
20
IC_{50} = 360 nM
[CI 303-427]
10^{-9}
10^{-8}
10^{-7}
10^{-6}
10^{-5}
[SBI-0206965] (mol.L-1)
B) Inhibition of human AMPK complexes
AMPK activity (% control)
100
80
60
40
20
α1β1γ1
IC_{50} = 247nM
[CI 199-306]
α2β2γ1
IC_{50} = 206 nM
[CI 177-239]
10^{-9}
10^{-8}
10^{-7}
10^{-6}
10^{-5}
[SBI-0206965] (mol.L-1)
C) Inhibition of human α2 kinase domain
AMPK activity (% control)
100
80
60
40
20
IC_{50} = 545nM
[CI 462-643]
10^{-9}
10^{-8}
10^{-7}
10^{-6}
10^{-5}
[SBI-0206965] (mol.L-1)
