# Supplementary material for: BAY-3827 and SBI-0206965: Potent AMPK Inhibitors That Paradoxically Increase Thr172 Phosphorylation
Source: Int J Mol Sci. 2023 Dec 29;25(1):453. doi: 10.3390/ijms25010453 (PMC10778976; doi:10.3390/ijms25010453)
Supplement: Supplementary file 1 [file ijms-25-00453-s001.zip › Supplementary Figure Legends.pdf]

SUPPLEMENTARY FIGURE LEGENDS

Figure S1: Selectivity of compound C, SBI-0206965 and BAY-8732 as kinase inhibitors. (A) Heat maps, generated using Microsoft Excel, were derived from screens of different human kinases using the indicated inhibitor. The key gives the color representing specified degrees of inhibition obtained, with concentrations of inhibitor and ATP used, and the number of kinases screened, shown on the left. The number of kinases screened differed for each of the three inhibitors, but the width of each data row has been kept constant to allow easy comparison. (B) degree of inhibition (± SD, n = 2) for the tenth percentile of kinases most potently inhibited by SBI-0206965. (C) degree of inhibition for the tenth percentile of kinases most potently inhibited by BAY-3827. Original data for compound C are from the MRC Kinase Inhibitor Database (Kinase-Profiling-Inhibitor-Database, 2023), for SBI-0206965 are from Ahwazi et al (2021) and for BAY-3827 are from Lemos et al (2021).

Figure S2: Structures of the AMPK inhibitors SBI-0206965 and BAY-3827, the control compound BAY-974, and the BAY-3827 lead compound, compound 6. Based on Egan et al (2015), Lemos et al (2021) and data in Lemos and Schulze (2023).

Figure S3: Effects of SBI-0206965 on preparations of purified AMPK in cell-free assays. (A) inhibition of rat liver AMPK by increasing concentrations of SBI-0206965. The filled circles are the mean ± SD of the actual data (n = 2), while the continuous line was obtained by fitting to the equation $Y=100-(100*X)/(IC_{50}+X)$ where IC_{50} is the concentration of SBI-0206965 giving a half-maximal effect; values for IC_{50} and 95% confidence interval (CI) are shown. (B) As (A) but using bacterially expressed human $\alpha1\beta1\gamma1$ or $\alpha2\beta2\gamma1$ complexes that had been maximally phosphorylated on Thr172 by CaMKK2. (C) As (A) but using bacterially expressed $\alpha2$ kinase domain (residues 1-310) that had been maximally phosphorylated on Thr172 by CaMKK2.

Figure S4: Lack of effect of (A) BAY-3827 and (B) SBI-0206965 on the activity of PPM1A assayed using the alternative model substrate p-nitrophenyl phosphate.
