# Supplementary figures and images for: BAY-3827 and SBI-0206965: Potent AMPK Inhibitors That Paradoxically Increase Thr172 Phosphorylation
Source: Int J Mol Sci. 2023 Dec 29;25(1):453. doi: 10.3390/ijms25010453 (PMC10778976; doi:10.3390/ijms25010453)

Figure S2

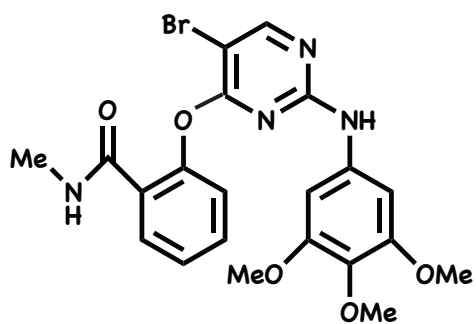

**SBI-0206965**

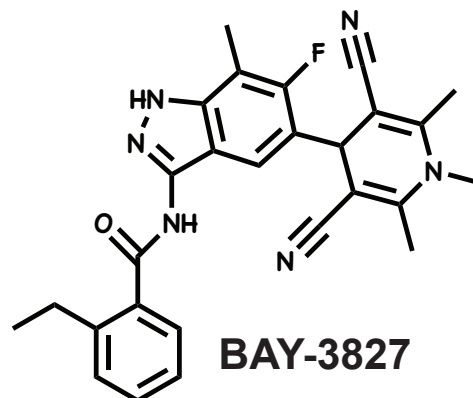

**BAY-3827**

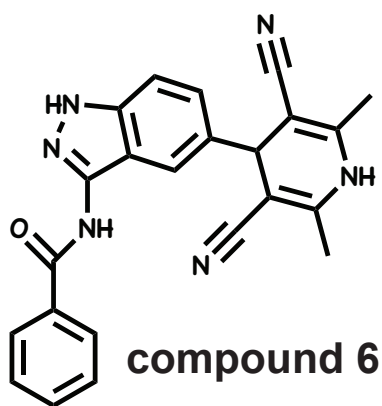

**compound 6**

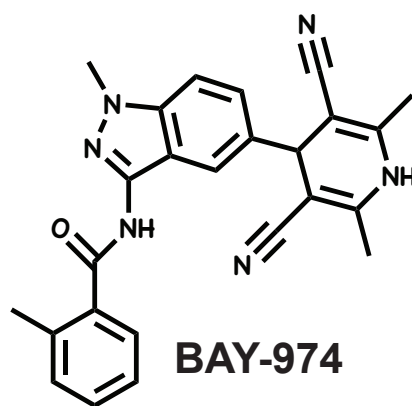

**BAY-974**

Supplement: Supplementary file 1 [file ijms-25-00453-s001.zip › FigS2.pdf]

Figure S4

A) Effect of BAY-3827 on PPM1A activity

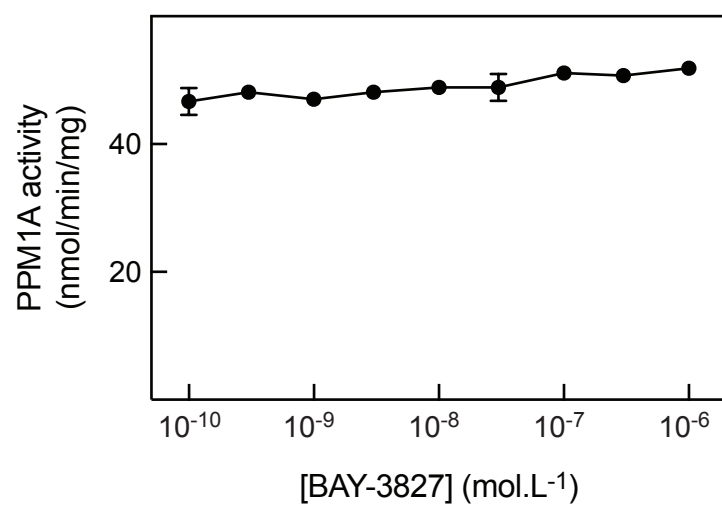

B) Effect of SBI-0206965 on PPM1A activity

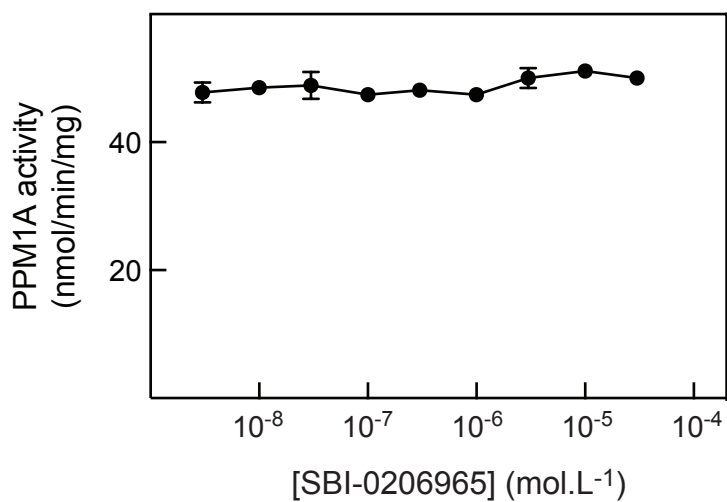

Supplement: Supplementary file 1 [file ijms-25-00453-s001.zip › FigS4.pdf]
